# Possible Involvement of Liver Resident Macrophages (Kupffer Cells) in the Pathogenesis of Both Intrahepatic and Extrahepatic Inflammation

**DOI:** 10.1155/2017/2896809

**Published:** 2017-07-19

**Authors:** Yuki Kakinuma, Takuya Kimura, Yoshifumi Watanabe

**Affiliations:** Department of Pharmaceutical Sciences, Musashino University, Tokyo 202-0023, Japan

## Abstract

Liver resident macrophages designated Kupffer cells (KCs) form the largest subpopulation of tissue macrophages. KCs are involved in the pathogenesis of liver inflammation. However, the role of KCs in the systemic inflammation is still elusive. In this study, we examined whether KCs are involved in not only intrahepatic inflammation but also extrahepatic systemic inflammation. Administration of clodronate liposomes resulted in the KC deletion and in the suppression of liver injury in T cell-mediated hepatitis by ConA as a local acute inflammation model, while the treatment did not influence dextran sulfate sodium- (DSS-) induced colitis featured by weight loss, intestinal shrink, and pathological observation as an ectopic local acute inflammation model. In contrast, KC deletion inhibited collagen-induced arthritis as a model of extrahepatic, systemic chronical inflammation. KC deleted mice showed weaker arthritic scores, less joint swelling, and more joint space compared to arthritis-induced control mice. These results strongly suggest that KCs are involved in not only intrahepatic inflammatory response but also systemic (especially) chronic inflammation.

## 1. Introduction

Kupffer cells (KCs) are liver resident macrophages which represent the largest population of macrophages in the body, constituting approximately 10% of all hepatic cells and approximately 80% of the tissue macrophages, including alveolar, splenic, and peritoneal macrophages [1]. KC has been a mysterious population for long time because the features including specific markers and the functions have been elusive, and only the designation “liver resident macrophage” has been established. Recently, some researchers tried to find and report the specific markers of the population [2–7]. These markers are becoming powerful tools to identify this population. Compared to specific markers, the functions have been extensively investigated, especially (and naturally) in the field of liver inflammation [8–14]. For these studies, one important finding by Van Rooijen and Sanders was the key triggering method that intravenous administration of clodronate-containing liposomes specifically deletes spleen and liver but not other tissue resident macrophages [15]. However, the attention of the researchers have been restricted only to the functions of Kupffer cells in the specific local site, liver. We speculated that this largest population of tissue macrophages has influence on systemic inflammation in some way. In this study, we examined the role of Kupffer cells in local, systemic, acute, and chronic inflammation models and indicate that Kupffer cells are involved in the pathogenesis of collagen-induced arthritis as a model of chronic, systemic inflammation.

## 2. Material and Methods

### 2.1. Animals

Female ICR, C57BL6 and DBA/2 mice (6 weeks old) were purchased from Tokyo Laboratory Animals Sciences Co., Ltd. (Tokyo, Japan). All mice were housed in a SPF facility. Water and food were available ad libitum. Each in vivo experiment for pathological models was conducted with 7–10 mice per group. All animal experiments were conducted in accordance with local institutional guidelines for the care and use of laboratory animals.

### 2.2. Animal Models

#### 2.2.1. ConA Hepatitis

ConA hepatitis was induced as described [16]. Briefly, ConA (10 mg/kg) in PBS was intravenously injected into the tail vein of ICR mic;, GPT and GOT in the plasma were measured 3, 12, and 24 h after treatment.

#### 2.2.2. Dextran Sulfate- (DSS-) Induced Colitis

Colitis was induced by the dietary administration of 4% DSS solution for 7 days via drinking water ad libitum and then back to water without DSS in C57BL/6 mice as described [17]. DSS was obtained from Wako Chemicals, Ltd. (Tokyo, Japan). Mice were inspected daily and the body weights were measured daily and they were scarified 10 days after the start of pathological inspection.

#### 2.2.3. Collagen-Induced Arthritis

CIA was induced by the administration of 100 ug collagen type II emulsified with complete Freund's adjuvant (70 ul) as the first immunization, followed by the second immunization with the same amount of collagen in incomplete Freund's adjuvant as described in detail [18]. The number of arthritic limbs were quantitated and each limb was assigned a severity score of 0–4 according to the criteria in [18].

### 2.3. Kupffer Cell Deletion

Kupffer cells were deleted from the liver by the intravenous administration of clodronate liposomes as described [19]. Clodronate liposomes were obtained from Katayama Chemicals, Ltd. (Osaka, Japan).

### 2.4. Cell Preparation

KCs were prepared by collagenase liver perfusion and differential density centrifugation. Briefly, the liver was perfused in situ with a HEPES based buffer containing 0.015% collagenase (Sigma) and 0.004% trypsin inhibitor (Wako Pure Chemical Industries Ltd., Osaka, Japan). After excision, the hepatic cells were suspended in Hanks' balanced salt solution and filtered through a nylon mesh. The filtrate was centrifuged thrice at 50 ×g (4°C) for 90 s to delete the parenchymal hepatocytes. Flow cytometric analysis involved centrifugation of purified fractions of nonparenchymal cells (NPC) from the liver, on a layer of 20% and 50% Percoll, at 1,000 ×g (4°C) for 20 min in order to remove dead cells. The suspension containing KCs was collected from the intermediate layer and resuspended in PBS containing 5 mM EDTA and 1% FBS, for antibody binding as described [7].

### 2.5. cDNA Synthesis, DNA Analysis, and RT-PCR

Total RNA was extracted from the cells using TRIzol Reagent (Invitrogen, Carlsbad, CA, USA), according to the manufacturer's instructions. Complementary DNA was synthesized using the SuperScript™ III Cells Direct cDNA Synthesis System (Invitrogen). Complementary RNA amplification, labeling, hybridization, and analysis, between the KCs and PEC macrophages, were performed by Toray Research Institute, Co. Ltd. (Kamakura, Japan). Saturation of PCR products was avoided using 20 PCR cycles during amplification. The PCR reactions were carried out using a three-step protocol: 94°C for 15 s, annealing at 55°C for 15 s, followed by 72°C for 30 s. Actin or GAPDH was used as an internal reference. The PCR products were analyzed by 2% agarose gel electrophoresis. The primers employed in the experiments are described [20].

### 2.6. Flow Cytometry Analysis

Nonspecific binding of immunoglobulin to the Fc receptors was blocked by preincubating 200 *μ*l of cell suspension, adjusted to 5 × 10^6^ cells/ml, with 25 ng/ml unlabeled murine polyclonal IgG antibody and 2.5 ng/ml anti-mouse CD16/32 antibody (BioLegend), at 4°C for 10 min. Cell surface molecules were stained with the primary antibody, followed by the labeled-secondary antibody or 2.5 ng/ml fluorescent-labeled antibodies, at 4°C for 20 min. After washing with ice-cold PBS, cells were resuspended in up to 300 *μ*l of sheath fluid containing 5 ng/ml propidium iodide, in order to identify the dead cells. Data acquisition and analysis were performed on the BD FACSAriaTM II flow cytometer (Becton, Dickinson and Company, Franklin Lakes, NJ, USA). The results in manuscript were the representatives of three or four experiments.

### 2.7. MTT Assay

The cell number of treated cells was evaluated by MTT assay [21]. Briefly, MTT was added to treated cells plated in a 96-well plate at a final concentration of 500 ug/ml, and the cells were incubated for 4 h at 37°C. Then 100 ul of acidic isopropyl alcohol was added to each well. The solution was vigorously mixed to solubilize the reacted dye. The absorbance of each well at 550 nm was measured using the microplate reader.

### 2.8. Histological Analysis

Tissues were dissected and fixed overnight in 4% paraformaldehyde solution at 4°C. Tissues were embedded in Tissue-Tek compound (Miles Laboratories, Elkhart, IN) and frozen in liquid nitrogen for preparation of cryostat sections. All sections (10 *μ*m) were lightly counterstained with 0.1% hematoxylin and eosin [22].

### 2.9. Statistical Analysis

The data are representative of three or four experiments. Experiments were performed in triplicate, and the error bars are the mean standard deviation (SD) of a single experiment. Differences between groups were examined for statistical significance using one-way ANOVA followed by the Student-Newman-Keul test. A *P* value of less than 0.05 denoted the presence of a statistically significant difference.

## 3. Results

### 3.1. Treatment with Clodronate Liposome Deletes Kupffer Cells but Does Not Affect Other Cell Populations

**Figure 1:**
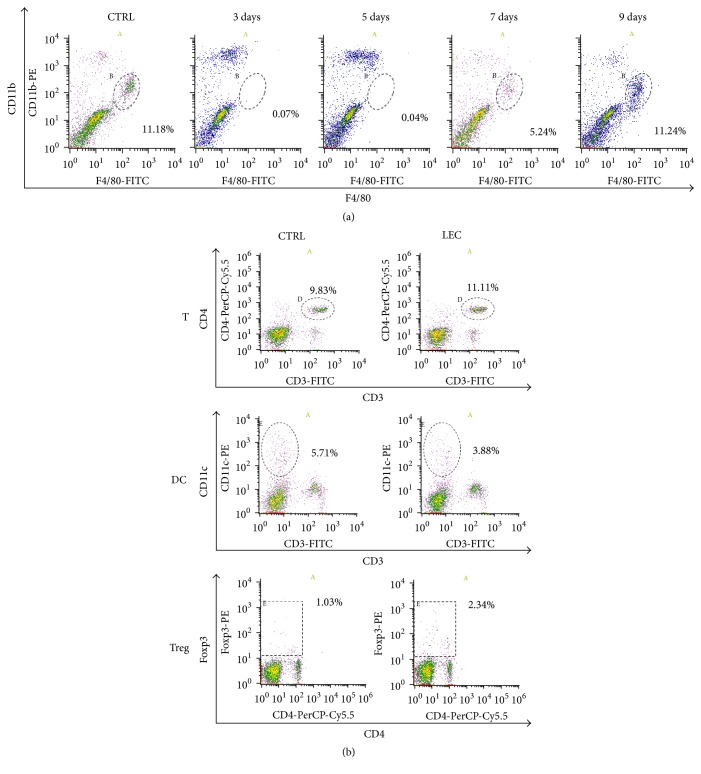
*Treatment with clodronate liposome deletes Kupffer cells but does not affect other cell populations*. DBA/2 mice were intravenously administered with 100 ul of clodronate liposome. (a) Nonparenchymal cells from the livers were collected by collagenase infusion described in Material and Methods at indicated days after treatment and then subjected to flow cytometry analysis using anti-F4/80 and anti-CD11b antibodies. The population of Kupffer cells was indicated as F4/80 positive and CD11b middle positive population. (b) Splenocytes from control or treated mice 3 days after treatment were collected and subjected to flow cytometry analysis for helper T cells (CD3^+^CD4^+^), dendritic cells (DC) (CD3^−^CD11b^+^), and regulatory T cells (CD4^+^Foxp3^+^). A, B, D, and E are target populations.

As already known, administration of clodronate-encapsulated liposomes deletes Kupffer cells (KCs) in vivo [15]. However, the effective duration time and influence on other cell types are not well understood. We first examined the duration time of KC deletion in the liver and the changes of other cell populations after treatment (Figure 1). The population of KCs was expressed as F4/80^+^CD11b^middle+^ group (Figure 1(a)) and the population was completely deleted by clodronate-liposome administration for 5 days after treatment and then gradually restored from 7 to 9 days (Figure 1(a)). In contrast, the treatment did not show any significant influence on the population of helper T cells (CD3^+^CD4^+^), DCs (CD3^−^CD11c^+^), or regulatory T cells (Foxp3^+^) (Figure 1(b)). These results show that clodronate liposomes induce selective but temporal deletion of KCs.

### 3.2. Deletion of Kupffer Cells Suppresses Local Acute Inflammation in the Liver

**Figure 2:**
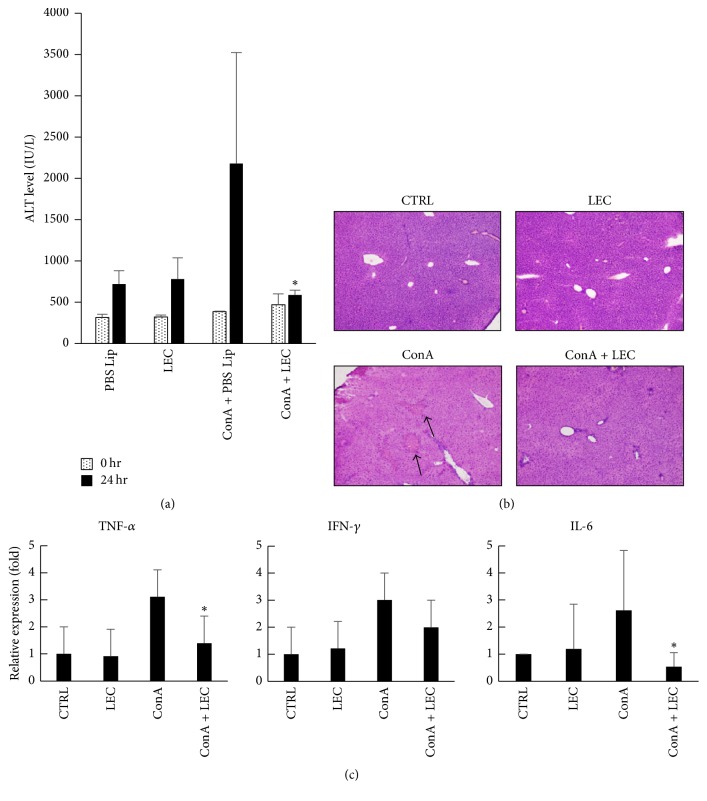
*Deletion of Kupffer cells suppresses ConA-induced hepatitis*. Con A (27.5 mg/kg) was intravenously injected into control or clodronate-liposome (Clp) treated mice 3 days after liposome treatment. Mice were dissected 24 h after ConA treatment and the ATL activities in the serum were measured (a) and the livers were subjected to histological analysis (b). Real-time PCR was performed using cDNA from the liver in the treated mice for the quantification of IFN-*γ*, TNF-*α*, and IL-6 mRNA. ^*∗*^*P* < 0.01.

To examine the role of KCs in local acute inflammation, we investigated T cell-mediated acute hepatitis model induced by concanavalin A (ConA). ConA induced liver injury within 24 h after administration (Figure 2(a)) and the deletion of KCs completely suppressed the hepatitis in the aspects of ALT and tissue section observation (Figures 2(a) and 2(b)). Deletion of KCs resulted in the inhibition of inflammatory cytokine mRNA expression such as IFN-*γ*, TNF*α*, and IL-6. In particular, suppression of TNF*α* and IL-6 mRNA expression were greater than IFN-*γ*. Thus, it is assumed that TNF*α* and IL-6 produced by KCs play a significant role in the pathogenesis of the hepatitis.

### 3.3. Kupffer Cells Are Not Involved in the Pathogenesis of Chemically Induced Extrahepatic Acute Inflammation

**Figure 3:**
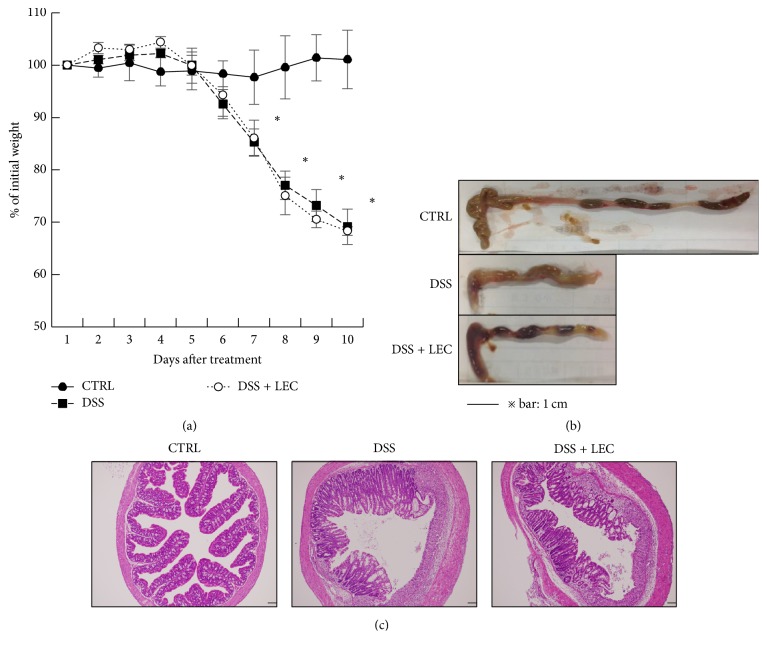
*DSS-induced colitis was not suppressed by the deletion of Kupffer cells*. C57BL/6 mice were treated with 100 ul of clodronate liposome (LEC) 24 h before DSS administration. The mice were administered 3% DSS in the drinking water ad libitum for 7 days and then back to water without DSS for 3 days. Mice were subjected to pathological analysis. (a) Daily weight changes of treated mice. (b) Comparison of intestines from treated mice. (c) Histological analysis of the samples in (b). CTRL: control; DSS: DSS treated; DSS + LEC: DSS treated in KC deleted mice. ^*∗*^*P* < 0.01.

Next, we examined the influence of KC deletion on dextran sodium sulfate- (DSS-) induced colitis as a model of extrahepatic acute inflammation, which shows features of ulcerative colitis with inflammation and exhibits features of Crohn's disease such as Th1 dependency [23, 24]. In this model system, KC deletion did not show any significant changes in the features (weight (Figure 3(a)), colon length (Figure 3(b)), and tissue histology (Figure 3(c))). Although DSS induced weight loss because of diarrhea with the shrink of colon, KC deletion did not inhibit the colon injury (Figures 3(a) and 3(b)). DSS also induced colon inflammation with multifocal submucosal oedema and pronounced loss of goblet cells (Figure 3(c)), which was not influenced by the administration of clodronate liposomes.

### 3.4. Kupffer Cells Are Involved in the Systemic Chronic Inflammation

**Figure 4:**
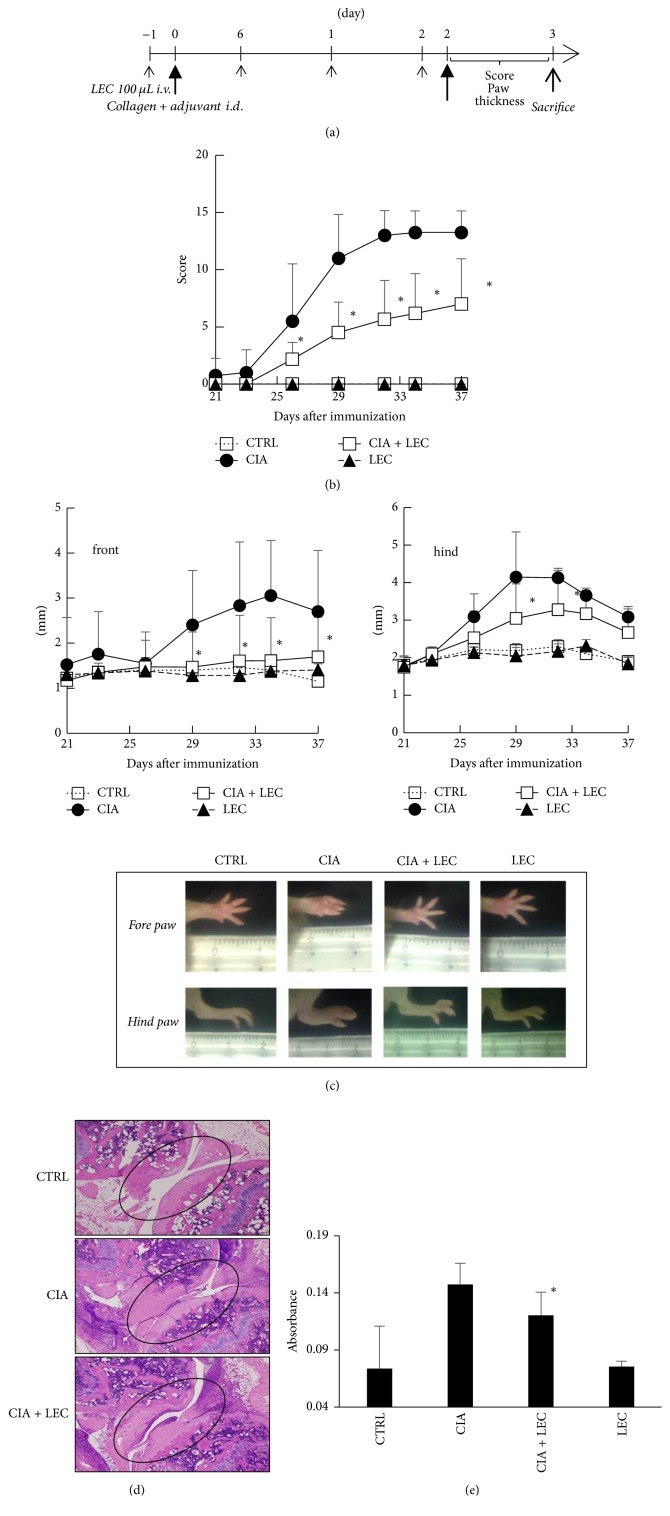
*Deletion of Kupffer cells partly regulated collagen-induced arthritis*. Collagen-induced arthritis was induced in control or KC deleted DBA/2 mice as scheduled in (a). One hundred ul of clodronate liposome (LEC) was administered every 7 days (small arrows). Collagen was intradermally injected at days 0 and 21 as middle arrows. (b) Severity scores of arthritic limbs from treated mice. The number of arthritic limbs was quantitated and assigned a severity score as described in Material and Methods. ^*∗*^*P* < 0.01 (c) Comparison of limbs thickness as the feature of arthritis. ^*∗*^*P* < 0.01 (d) Histological analysis of the limb joints from the treated mice. (e) Splenocytes response activity assay. Splenocytes from the sacrificed mice were stimulated in vitro with collagen (75 ug/ml) for 72 h and then MTT assay was performed. ^*∗*^*P* < 0.05.

Finally, we examined the role of KCs in collagen-induced arthritis (CIA) as a systemic chronic inflammation model. Different from chemically induced acute colitis, collagen-induced chronic arthritis is mediated by acquired immune reaction [18], which require various kinds of cell types for establishment. The score of arthritic feature increased after the collagen second challenge (Figure 4(b)). In contrast, KC deletion significantly suppressed the score (Figure 4(b)). In terms of paw swelling, the same tendency was observed in both front and hind paws (Figure 4(c)). Histological features also confirmed the above observation. As shown in Figure 4(d), collagen challenge induced joint swelling and the joint space was scarcely observed compared to control. In contrast, KC deletion suppressed the joint swelling and maintained the joint space (Figure 4(d)). In order to examine the T cell response as the establishment of acquired immunity, the splenocytes from collagen-challenged mice were collected 15 days after the challenge and then stimulated with collagen in vitro. Three days after the in vitro stimulation, the cell number of the samples was measured by MTT assay.  Figure 4(e) shows that splenocytes from KC deleted mice challenged with collagen exhibited less proliferation ability compared to those from collagen-challenged mice. KC deletion without collagen challenge did not show any significant change as control.

## 4. Discussion

In this study, we show that Kupffer cells (KCs) are involved in the onset of both local (acute hepatitis) and systemic (collagen-induced arthritis) inflammation.

Several studies reported that clodronate liposomes (CLs) suppressed arthritis when prepared with small unilamellar vesicles (SUV) and administered systemically postarthritis induction [25, 26] or administered directly into joints [27, 28]. These studies suggest the involvement of synovial tissue macrophages of joints in the progression of arthritis. Both routes of CLs administration deleted or decreased the synovial macrophages in joints and the reduction was correlated with the suppression of arthritis [25–28]. However, systemic administration of CLs did not influence the number of synovial phagocytic cells in normal [15] or inflammatory joints when the liposomes were prepared with multilamellar vesicles (MLV) [25]. Commercially available reagent used in this study is composed of MLVs; thus, the reagent is thought not to influence synovial macrophages in joints. In contrast, it is thought that when the liposomes are prepared with SUV, these nanoparticles could pass through the endothelial fenestrae of blood vessels only in the inflammatory tissues because of enhanced permeability and retention (EPR) effect [29] by cytokines such as VEGF (also designated Vascular Permeability Factor (VPF) because of the function), the major cytokine to induce blood vessel permeability, in inflammation [30]. In fact, it is reported that SUV-CLs are more effective than MLV-CLs to decrease the number of synovial macrophages in inflammatory joints [26]. However, these reports suggest the significance and role of synovial macrophages in joints but not those of KCs which were deleted at the same time. To examine the role of KCs, CLs prepared with MLV to avoid the influence on synovial macrophages should be administered before the onset of arthritis induction.

At present, we do not have any concrete data to explain how KCs are involved in the pathogenesis of arthritis. Basically, KCs are heterogeneous cell populations [31, 32] and show inflammatory phenotypes (M1) in some cases [9, 11, 12, 14, 33] while they do anti-inflammatory phenotypes (M2) in others [34–38], depending on the circumstance conditions [10]. In this study, KCs played as M1 cells in the onset of arthritis. Phagocytic cells in the synovial membrane of joints are thought to be critical mediators in the inflammation of arthritis according to the previous studies [26–28]. Synovial macrophages comprise two subsets: resident tissue macrophages and infiltrated inflammatory macrophages. It is noteworthy that the phagocyte population involved in the pathogenesis of arthritis expresses complement receptor for C3b and iC3b (CRIg) (also designated VSIG4 or Z39Ig) [39, 40], which is selectively expressed on tissue macrophages, mainly on KCs [3, 4], and the expression increased according to the severity of the arthritis [40]. The antigen itself functions as anti-inflammatory by inducing tolerance in T cells rather inflammatory [4]. Thus, it is thought that the increase amount of antigen reflects the increase number of KC-like cells in the synovial membrane of joints. Some studies suggest the similarity of the phagocytic cells in synovial membranes of arthritis joints to KCs in the liver [39, 40]. Alternatively, KCs may increase the number of these synovial phagocytes. Thus, it is assumed that the interaction between KCs and these phagocytic cells in the joints is the key of the mechanism. The possible hypothesis is that cytokine(s) from KCs activates distal phagocytic cells in synovial membranes of joints. Such cytokine(s) is not elucidated, but IL-4 to induce the proliferation of tissue macrophages is a potential candidate [41], or it is suggested that there is a network between KCs and monocytes, in which different macrophage populations communicate, activate, or suppress each other [42]. Recently, Fujiu et al. reported that the existence of homeostasis regulation between distal organs (heart-brain-kidney) through the tissue macrophage communication [43]. Although KCs are fully capable of migrating in vitro [7], it is not naturally thought that KCs themselves migrate into joints and induce inflammation. So far, we cannot completely delete the possible involvement of splenic or synovial macrophages in the regulation in spite of the fact that the major population which MLV clodronate liposomes delete is KCs. It is difficult to identify the substantial specific tissue macrophage population because there is not any marker with strictly restricted expression on the specific population of tissue macrophages. This issue is to be solved.

More possible explanation is that KCs promote Th17 differentiation. IL-17 producing T helper cells, Th17 cells, are key cell population in the pathogenesis of various autoimmune and inflammation diseases [44], including autoimmune arthritis [45, 46], and these cells play a central role in collagen-induced arthritis [47]. The differentiation of these cells is dependent on the copresence of IL-6 and TGF-*β* [44]. Interestingly, Kupffer cell is the main cell population to produce TGF-*β* in normal liver [48, 49] and these cells also express IL-6 by the stimulation of inflammation. Thus, it is naturally thought that KCs promote Th17 differentiation. In fact, Xie et al. reported that KCs induce Th17 differentiation at local site (liver). Considering the large size of KC population, these cytokines are thought to regulate the Th17 differentiation in distal sites. Sasai et al. reported that IL-6 promoted the onset of CIA [50]. The hypothesis that KCs promote Th17 differentiation in synovial joints by producing TGF*β* and IL-6 is under investigation.

Deletion of KCs did not suppress DSS-induced colitis in all features of colitis (Figure 3). DSS-induced colitis lacks involvement of the adaptive immune system in the pathogenesis of the inflammation [24]; however, gut macrophages are thought to be main mediators because gadolinium chloride (GdCl_3_), a macrophage selective inhibitor, ameliorated the mucosal damage by suppressing DSS-induced macrophage-derived cytokines (IL-6, IL-1*β*, and TNF*α*) [51]. According to the results in this study, KCs do not interact with these macrophages. The detail mechanism is to be examined.

In conclusion, KCs are involved in both intrahepatic liver inflammation and extrahepatic pathogenesis of chronic arthritis, but not acute extrahepatic colitis. KCs are thought to regulate the chronic inflammation of arthritis probably through the synovial membrane phagocytes.

## 5. Conclusions

In this report, we show the possibility that liver resident macrophages (Kupffer cells), being considered to be involved in only specific local liver inflammation, are involved in extrahepatic inflammation. This finding will shed novel light on the role of this cell population in the pathogenesis of systemic diseases.
